# Managing healthcare for female BRCA carriers in the population screening era: developing a harmonized national policy for surveillance and risk-reduction

**DOI:** 10.1186/s13584-026-00746-3

**Published:** 2026-01-30

**Authors:** Rachel Michaelson-Cohen, Shunit Armon, Naama Srebnik, Einat Koller Volkow, Pnina Mor, David Gekhtman, Ora Rosengarten, Adi Maisel Lotan, Hadar Goldvaser, Yahelli Miller, Dana Madorsky Feldman, Rinat Bernstein Molho, Miri Sklair Levy, Inbal Kedar, Yael Goldberg, Yael Raz, Sharon Simchoni, Aviad Hoffman, Miora Linial, Einat Carmon, Inbar Gatot, Michal Braha, Rakefet Chen Shtoyerman, Shikma Mordechai, Elizabeth E. Half, Lior Katz, Zohar Levi, Sharon Bratman Morag, Tanir M. Allweis, Eitan Friedman, Ephrat Levy-Lahad, Sari Lieberman

**Affiliations:** 1 The Faculty of Medicine, The Hebrew University of Jerusalem, Jerusalem, Israel; 2 The Fuld Family Medical Genetics Institute, The Eisenberg R&D Authority, Shaare Zedek Medical Center, Jerusalem, Israel; 3 Department of Obstetrics and Gynecology, The Eisenberg R&D Authority, Shaare Zedek Medical Center, Jerusalem, Israel; 4 Department of Imaging, Shaare Zedek Medical Center, Jerusalem, Israel; 5 Gyneco-oncology unit, Helmsley Oncology Center, Shaare Zedek Medical Center, Jerusalem, Israel; 6 Department of Plastic and Reconstructive Surgery, Shaare Zedek Medical Center, Jerusalem, Israel; 7 Breast Medicine Service, Department of Medicine, Memorial Sloan Kettering Cancer Center, NY, USA; 8 Department of General Surgery, Shaare Zedek Medical Center, Jerusalem, Israel; 9 Meirav High Risk Clinic, Sheba Medical Center, Tel Hashomer, Israel; 10 Susanne Levy Gertner Oncogenetics Unit, The Danek Gertner Institute of Human Genetics, Sheba Medical Center, Ramat Gan, Israel; 11 Gray Faculty of Medical and Health Sciences, Tel Aviv University, Tel Aviv, Israel; 12 Breast Imaging Unit, Division of Diagnostic Imaging, Sheba Medical Center, Tel Hashomer, Israel; 13 Recanati Genetics Institute, Rabin Medical Center, Petach Tikva, Israel; 14 Department of Obstetrics and Gynecology, Tel Aviv Sourasky Medical Center, Tel Aviv, Israel; 15 Genetics Institute and Genomics Center, Tel Aviv Sourasky Medical Center, Tel Aviv, Israel; 16 Department of General Surgery, Rambam Health Care Campus, Haifa, Israel; 17 Ruth and Bruce Rappaport Faculty of Medicine, Technion Israel Institute of Technology, Haifa, Israel; 18 Medical Genetics Unit, Department of Obstetrics and Gynecology, Assuta Ashdod Medical Center, Ashdod, Israel; 19 Department of General Surgery, Assuta Ashdod Medical Center, Ashdod, Israel; 20 Division of Surgery, Sackler Faculty of Medicine, Assaf Harofeh Medical Center, Tel Aviv University, Tel Aviv, Israel; 21 Department of Molecular Biology, The Adelson School of Medicine, Ariel University, Ariel, Israel; 22 The Genetics Institute, Kaplan Medical Center, Rehovot, Israel; 23 Genetics Unit, Rebecca Sieff Hospital, Safed, Israel; 24 Department of Gastroenterology, Rambam Health Care Campus, Haifa, Israel; 25 Department of Gastroenterology, Hadassah Medical Center, Jerusalem, Israel; 26 Division of Gastroenterology, Rabin Medical Center, Petach Tikva, Israel; 27 BRCA Clinic, Rambam Health Care Campus, Haifa, Israel; 28 Genetics Institute, Rambam Health Care Campus, Haifa, Israel; 29 Department of Breast Surgery, Hadassah Hebrew University Medical Center, Jerusalem, Israel; 30 Oncogenetics Service, Assuta Medical Center, Tel Aviv, Israel

**Keywords:** Hereditary breast and ovarian cancer, BRCA-PV carriers, Cancer risk reduction, High risk surveillance clinic, National protocol

## Abstract

**Background:**

The implementation of Israeli National screening for *BRCA1/BRCA2* pathogenic variants (PVs) in 2020 has led to a significant increase in the number of unaffected female carriers who are referred to high-risk surveillance clinics (HRSCs). Lack of standardization in protocols for risk reduction and surveillance between HRSCs results in confusion and gaps in care. We aimed to identify discrepancies in existing practices and lead to policy development of a national policy for surveillance, management and risk reducing strategies in *BRCA-PV* carriers.

**Methods:**

A comparative analysis of risk reduction and surveillance protocols of the nine leading HRSCs across Israel, comprising the Israeli Consortium for hereditary breast and ovarian cancer (HBOC), and multi-center meetings to develop consensus guidelines for HRSCs.

**Results:**

Our analysis revealed a high level of prior consensus on critical aspects including risk-reducing mastectomy, salpingo-oophorectomy, fertility treatment, contraception, hormone replacement therapy, and general health behavior. For breast cancer (BC) imaging surveillance there was variability regarding frequency (e.g. only one HRSC offers biannual MRI for *BRCA1* carriers), age limits (five centers continue in women older than 75 years), frequency during pregnancy and lactation (four HRSCs every three months and four others every six months; one does not recommend any surveillance), and surveillance post-mastectomy. For ovarian cancer (OvCa) surveillance, there was also variability: six centers recommend biannual/annual serum CA-125 level and pelvic sonography for all women, one center recommends this for all women till risk-reducing-bilateral-salpingo-oophorectomy (RRBSO), and two centers exclusively for women from age 35 till RRBSO. Surveillance recommendations for malignancies other than BC and OvCa differed greatly among centers.

**Supplementary Information:**

The online version contains supplementary material available at https://doi.org/10.1186/s13584-026-00746-3.

## Background

Breast and ovarian cancer (OvCa) are health concerns worldwide. Among Israeli women, breast cancer (BC) is the most common malignancy, and leading cause of cancer death, while OvCa is the most lethal gynecological cancer [1, 2]. Combined, they were the leading cause of death in Israeli women aged 25–84 in 2020, with a mortality rate [1]. Among Ashkenazi Jews (AJs), 1:40 is a carrier of one of three recurring *BRCA1*/*BRCA2* pathogenic variants (PVs) [3, 4] and 10% of breast cancer (BC) and 40% of OvCa are related to germline *BRCA*-PVs [5–7]. Given the substantially elevated lifetime risk of developing BC and OvCa among *BRCA-*PV carriers (PVCs) [3], they are advised to undergo enhanced surveillance and risk-reducing procedures, such as risk-reducing mastectomy (RRM), which reduces morbidity, and risk-reducing-bilateral-salpingo-oophorectomy (RRBSO), which reduces morbidity and all-cause mortality [8, 9].

Traditionally, *BRCA1/2* testing was offered to women affected with BC/OvCa, women with significant family history (FH) of cancer, or individuals with familial *BRCA-*PVs following genetic counselling (GC) [10]. However, approximately half of BRCA-PVCs lack this background and are identified after cancer diagnosis [5]. Recognizing these limitations and based on cost-effectiveness of population screening (PS) [11, 12], Israel became the first country globally to initiate free PS for all women with AJ ancestry [13]. *BRCA-*PVC*s* are referred to high-risk surveillance clinics (HRSCs) after genetic counseling.

PS Implementation has led to a significant increase in newly identified *BRCA*-PVCs. From mid-2021 through 2024, approximately 2,350 of 165,000 (1.42%) women tested via PS were identified as carriers (~ 650 annually). Based on the experience of the Shaare Zedek Medical Center (SZMC) HRSC, since 2021 most of newly identified *BRCA-*PVCs were identified by PS rather than through Oncogenetics clinics. The increase in newly diagnosed *BRCA-*PVC*s* seeking HRSCs has led to an overflow of many existing clinics, and establishment of new HRSCs in hospital and community-based settings.

A recently published study aiming to determine the availability and practices of HRSCs in Israel, identified nine clinics managing approximately 4,500 *BRCA-*PVCs, with different surveillance guidelines [14]. Most centers independently developed their own protocols based on international guidelines, such as the National Comprehensive Cancer Network (NCCN, USA) and European Society for Medical Oncology (ESMO) [9, 15]. However, these guidelines differ in certain areas, such as breast imaging and OvCa surveillance, as described by comparative analyses of international protocols [16, 17]. Differences in guidelines are attributed to various factors, including insufficient evidence level for certain recommendations, cost–benefit analyses, risk perception variations and acceptability of risk-reducing strategies.

Standardized guidelines are essential for ensuring consistency of care, and for avoiding patients receiving conflicting recommendations from different centers. To this end, we evaluated current practices across HRSCs in Israel, emphasizing controversial areas. Our goal was to create an updated foundation for development of national guidelines for management of *BRCA-*PVCs in Israel.

## Methods

The HRSC team at SZMC conducted a comprehensive review of *BRCA* carrier management protocols. A multidisciplinary panel was convened to evaluate all aspects of care including medical genetics, breast and reconstructive surgery, radiology, oncology, gynecology, reproductive endocrinology and gastroenterology. The panel developed recommendations based on current international guidelines, specifically National Comprehensive Cancer Network (NCCN, USA, 2025) and European Society of Medical Oncology (ESMO,2022), as well as consensus discussions addressing controversial areas.

We reached out to the other eight largest HRSCs in Israel, collectively managing approximately 4,500 *BRCA-PVCs*. Each clinic was requested to submit their surveillance protocol (as of 3.2025). A comparative analysis of these protocols was performed, identifying areas of discrepancy. We focused on breast imaging age and modalities, as well as imaging at different life stages. We addressed recommended age for risk-reducing surgeries, and various surgical options. Additionally, recommendations for surveillance for malignancies other than breast or OvCa were examined.

Discrepancies were discussed in multi-center meetings. A harmonized protocol was formulated based on HRSC protocols, aligned with NCCN ESMO guidelines. Consensus was reached upon agreement of at least 8 of 9 HRSCs (including SZMC).

## Results

High rates of consensus were noted on clinically critical aspects including recommendations for RRM, RRBSO, fertility treatments and preservation, oral contraception, hormone replacement therapy (HRT) and health behaviors. Detailed surveillance recommendations of the nine HRSC are summarized in Table 1. The complete protocol for *BRCA* carrier management as agreed upon by the Israeli consortium after data analysis and multi-center discussions, are found in Supplement 1, and our main recommendations are shown in Table 2, as compared with the international guidelines.

### Comparison of Israeli HRSCs

Inter-clinic variations were noted in recommendations pertaining to breast MRI frequency, age-specific criteria for imaging, BC surveillance during pregnancy and lactation, post-RRM surveillance, and approaches to OvCa screening and risk-reduction surgeries, such as early salpingectomy with delayed oophorectomy, and hysterectomy in addition to RRBSO. Additionally, recommendations regarding screening for non-BC/OvCa cancers differed between clinics. The following sections discuss different approaches for each topic, and propose consensus recommendations agreed upon in multi-center discussion. Unless otherwise noted, our recommendations are similar to those of the NCCN/ESMO.

**Areas of controversy**.

1. ***Breast imaging***:
  A. **Breast imaging modality**,** frequency and timing**:

*Background*:

Modality and frequency: several guidelines recommend alternating annual MRI and mammography (MG) at 6-month intervals for women with > 20% lifetime BC risk, including *BRCA-*PVC*s* [18]. There is potential harm from MG in young women due to ionizing radiation exposure [19], while MRI poses no radiation risk [19, 20]. Dynamic contrast-enhanced MRI (DCE-MRI) shows higher sensitivity and specificity than MG alone, detecting cancers earlier [21]. Both MRI and MG can cause anxiety from false positive results [22] and are associated with substantial costs, though annual MRI is highly cost-effective [23].A recent longitudinal multicenter cohort study revealed significantly reduced BC mortality (5.5% vs. 20.5%, *P* < 0.001) among women adhering to MRI surveillance compared to those who did not [24]. An Israeli retrospective study found limited additional benefit from annual ultrasound (US) when combined with MG and MRI screening in women aged 30–39 [25]. Regarding increased MRI frequency, a prospective study evaluating biannual MRI in addition to annual MG found that MRI detected invasive cancers < 1 cm in diameter, effectively avoiding interval invasive cancers, with low recall rates. Annual MG did not demonstrate screening benefit when performed in conjunction with biannual MRI [26].

Age limits: The optimal imaging initiation age (25 vs. 30 years) and upper age limit remain unresolved. BC risk in carriers between age 20–30 years is estimated as < 4% [3, 27]. For women aged 71–80, the residual risk is 7.2% and 8.8% for *BRCA1* and *BRCA2* carriers, respectively [3]. In an Israeli cohort of 88 participants with a mean age of 73.7 years, ten carriers (11.3%) were diagnosed with BC after age 70 [25].

#### International guidelines:

ESMO guidelines recommend biannual MRI for BRCA1 if available. Otherwise, US and MG may be considered in the interval between annual MRI, for carriers under and over 40 years of age, respectively. For BRCA2 PVCs, recommendations include annual MRI [28]. Screening is recommended from age 30 or 5 years before youngest family member's BC diagnosis. Upper age limits should be individualized based on breast density, comorbidities, and patient preference [15]. 

NCCN guidelines [9] recommend clinical breast examination (CBE) every 6–12 months, starting at age 25. Breast imaging recommendations are as follows: age 25–29 years, annual breast MRI (or MG, if MRI unavailable) or individualized based on FH of BC diagnosis before age 30; age 30–75 years, annual MG and breast MRI; from 75 years on, management individualized according to life expectancy and comorbidities.

#### Survey of HRSCs in Israel:

Most HRSCs recommend an annual MRI from age 25; annual breast US starting at an age 18–30 years in the interval between annual MRIs; annual MG from age 25–40. One center performs biannual breast MRI for *BRCA1* carriers. Five HRSCs recommend routine CBE every 6–12 months as part of their surveillance program: one for ages 25–30 only, and the other four for all ages. While the upper limit of screening in the general population is 75, HRSCs in Israel did not specify an upper age limit for BC screening [14].

#### Final Recommendation:

Age 25–29: MRI and CBE annually; Age 30–75: MRI and CBE annually, alternating with MG at 6-month intervals. For age 30–40, consider one view MG (MLO-mediolateral oblique) and interval MRI. Over 75: if there are no significant co-morbidities, following discussion with the patient, screening should be continued in the same manner.

B. **Breast imaging in pregnant and lactating women**.

*Background: *Pregnancy-associated BC (PABC) is defined as BC diagnosed during pregnancy, within the first-year post-partum, or at any time during lactation.

*BRCA2* carriers demonstrate transiently elevated BC risk following pregnancy, particularly within a two-year postpartum window [28]. Long-term risk is not elevated, with a protective effect observed in *BRCA1* carriers who have had at least four deliveries (OR = 0.62; 95%CI = 0.41–0.94) [29].Lactation decreases long-term BC and OvCa risk in the general population, as well as in *BRCA1/2* carriers [30, 31].

Despite the possible protective effect of pregnancy and lactation, these periods present a challenge for BC surveillance. The augmented breast tissue and vascular flow cause increased mammographic density and MRI background parenchyma enhancement, which impede test interpretation. Breast DCE-MRI is contraindicated during pregnancy due to concerns regarding fetal gadolinium exposure [32]. While DCE-MRI is safe during lactation, its efficacy remains uncertain [33]. Protocols for breast MRI during lactation demonstrate inconsistency, with some centers avoiding it.

A retrospective study of 142 MRI examinations during lactation demonstrated diminished overall specificity and increased BI-RADS 3 classifications [34]. A study of hundreds of US examinations performed every 3 months during pregnancy and lactation, as practiced in some Israeli centers, found a positive predictive value of 11% and 8%, respectively, for BC detection [29].

#### International guidelines:

The NCCN and ESMO guidelines have no specific recommendations for surveillance during pregnancy and breastfeeding. 

#### Survey of HRSCs in Israel:

Significant variability exists in this category. Three centers perform quarterly breast US while five others perform it biannually, with or without concurrent CBE. One center does not recommend surveillance for pregnant or lactating women. Among centers that defer MRI during lactation, considerable variation exists regarding the recommended interval between lactation cessation and resumption of MRI surveillance, ranging from 2 to 6 months. 

#### Final Recommendation:

During pregnancy: breast US every 3–6 months. During lactation: A discussion regarding risks and benefits should be held with women, including planned duration of breastfeeding. Mammography (to detect macrocalcifications) and MRI can be considered three months after delivery for women above 30, and especially above 35 years. The Ultrafast dynamic MRI protocol may assist in discrimination between prominent background parenchymal enhancement associated with lactation, and BC. Adherence to screening recommendations and ensuring time windows that allow imaging is important, especially in women with successive pregnancies, to avoid interval BC during the high-risk childbearing period.

C. **Breast imaging for women after RRM**.

*Background*: There is no clear radiologic definition of what constitutes substantial residual postoperative breast tissue [35], and therefore, whether post-RRM carriers should continue BC surveillance. The annual incidence of BC post-RRM is estimated as 0.08% and 0.21% in BRCA1 and BRCA2, respectively(36) which is lower than in the general population [37, 38]. 

#### Survey of HRSCs in Israel:

HRSCs recommend surveillance according to residual breast tissue volume. When minimal residual tissue is present by MRI, seven clinics recommend breast US (every 1-2 years), and/or CBE (every 6-12 months), among which two clinics also recommend an MRI every two years. The remaining two did not specify any recommendation in their protocol. In cases of “significant” residual breast tissue, most clinics in Israel recommend annual US or MRI [14].

#### Final Recommendation:

If substantial amount of residual tissue remains following surgery (based on MRI performed in the first year following surgery), MRI surveillance should be continued, and repeat surgery discussed [15]. If there is no substantial residual tissue, annual CBE is recommended, and US can be considered.

2. ***Ovarian cancer risk reduction and surveillance***:

The main areas of controversy include adding hysterectomy to RRBSO, Risk-reducing early salpingectomy and delayed oophorectomy (RRESDO), and surveillance before and following RRBSO.

A. **Adding Hysterectomy during RRBSO**:

*Background*: Adding hysterectomy to RRBSO for *BRCA* carrier remains controversial. Endometrial cancer (EC) risk, HRT implications, tamoxifen treatment in carriers affected with BC, and additional morbidity associated with a more protracted surgical procedure are issues to be weighed when considering hysterectomy. Whether risk for EC, particularly uterine serous papillary cancer (USPC), is elevated in *BRCA1*-PVCs is controversial, and in any case the absolute risk is low: about 3% and 1% for EC and USPC, respectively [39–41]. However there may be an advantage to hysterectomy with respect to HRT use in *BRCA*-PVCs [42–47].

Nevertheless, questions about the safety of HRT with regards to BC risk have been raised in the literature, including a study by our group which showed mildly increased risk when initiating HRT use over 45 years [48]. An observational study of *BRCA1* carriers post-RRBSO showed no increased BC risk with HRT overall, but found that combined estrogen-progesterone therapy conferred significantly higher risk than estrogen alone [49]. This is consistent with large studies in non-carriers showing that BC risk is increased when HRT regimens include progesterone. Hysterectomy is therefore advantageous in this respect, as it allows safer estrogen-only HRT [46]. For tamoxifen users, hysterectomy eliminates the increased risk of EC. Regarding surgical complications, compared to RRBSO hysterectomy is associated with several potential complications, including bleeding, infection, urinary tract injury, vaginal vault dehiscence and bowel injury [50, 51].

#### International guidelines:

ESMO guidelines do not mention hysterectomy as part of RRBSO for BRCA-PVCs, while the NCCN advises that risks and benefits of hysterectomy be discussed with BRCA-PVCs prior to RRBSO. 

#### Survey of HRSCs in Israel:

hysterectomy is not listed in recommendations [14]. A recent survey of 530 BRCA Israeli carriers demonstrated that while RRBSO was discussed with 91% of carriers, HRT was discussed with 59%, and the option of hysterectomy with only 27%, emphasizing the need for including these issues in presurgical consultation [52].

#### Final Recommendation:

routine hysterectomy during RRBSO for BRCA-PVCs is not recommended, as the absolute EC risk is low. However, hysterectomy should be discussed with carriers planning RRBSO. Factors to discuss are gene involved (BRCA1 vs. BRCA2), age at surgery, potential use of estrogen-only HRT, BC history and tamoxifen treatment, additional EC risk factors, and patient’s surgical risk (age, body mass index, history of pelvic surgery etc).

B. **Early salpingectomy and delayed oophorectomy**.

*Background*: Insights into the pathogenesis of high-grade serous OvCa have emerged from investigating occult carcinomas in BRCA1/2 PVCs, suggesting the fallopian tube as the origin of OvC [53]. Although RRBSO has been shown to reduce morbidity and overall mortality(8), premenopausal RRBSO leads to premature surgical menopause, which has detrimental long-term health sequelae, particularly if women are unable to use HRT [54]. Despite its proven benefits on morbidity and mortality, ~30% of BRCA-PVCs decline or delay RRBSO [8, 55]. RRESDO at age 35 with delayed oophorectomy has been suggested as a menopause-delaying procedure for BRCA-PVCs. In the general population, opportunistic salpingectomy reduces OvCa risk by 35-80%, without increased surgical morbidity [56, 57]. RRESDO is cost-effective and highly acceptable by BRCA-PVCs [58, 59]. A multicenter non-randomized trial demonstrated that women choosing RRESDO had fewer menopausal symptoms than women undergoing RRBSO, even among HRT users [60]. Of note, there is still no data demonstrating that RRESDO is associated with reduced OvCa risk in the interval between salpingectomy to oophorectomy. Clinical trials assessing the efficacy of RRESDO on reducing OvCa risk are currently ongoing (e.g. NCT02321228- registration date 2014-12-11, NCT01907789- registration date 2013-07-18, NCT04294927- registration date 2020-03-01).

#### International guidelines:

According to NCCN and ESMO guidelines, RRESDO is not routinely recommended 

#### Survey of HRSCs in Israel:

currently, RRESDO is not recommended in Israel as a preventive strategy [14], although it is occasionally performed on an individual basis.

#### Recommendation:

Until more data becomes available, RRESDO should not be routinely offered, and should be performed exclusively within clinical trials.

C. **OvCa surveillance before and after RRBSO and for women who decline RR surgery**:

*Background*: Available surveillance measures based on CA-125 and transvaginal US have not been shown to improve survival, neither in the general population [61], nor in BRCA-PVCs [61-62]. Surveillance is therefore not recommended by any national guidelines, and the focus is thus on surgical risk-reduction, rather than early detection. 

Despite RRBSO’s established efficacy, ~ 30% of *BRCA* patients decline surgery [8, 55].A recent Israeli study demonstrated that in PVCs who forgo RRBSO, awareness of *BRCA* PV prior to OvCa resulted in earlier stage and improved disease-free survival compared to post diagnosis genetic testing, though overall survival was not improved [63]. These findings were similar to those of a prior study showing advantage of surveillance amongst those forgoing RRBSO [64]Thus, while RRBSO is the universally adopted and most effective risk-reducing strategy, it remains possible that patients who decline surgery might derive some benefit from a surveillance program.

OvCa surveillance before surgery: *BRCA*-PVCs up to age 35 years are at low risk for OvCa. A large international study reported no OvCa cases below age 30, and the cumulative risk from age 30 to 40 was 2% in *BRCA1* with no cases in *BRCA2* [3]. In the Israeli population study, no OvCa cases were reported under age 40 [3, 27].

OvCa surveillance after surgery: In a recent cohort study following 6310 women after RRBSO, 20-year cumulative risk for primary peritoneal cancer (PPC) was 2.7% and 0.9% for *BRCA1* and *BRCA2*, respectively [65]. PPC risk factors include older age at RRBSO and findings of serous tubal intraepithelial carcinoma (STIC) in RRBSO specimens [66, 67].

#### International guidelines:

ESMO recommends considering surveillance with pelvic US and CA-125 biannually starting at age 35, until RRBSO is carried out. If carried out, surveillance should be provided in tertiary centers under structured protocols by experienced sonographers, and women should be counseled regarding screening limitations. NCCN guidelines recommend CA-125 testing and pelvic US exclusively for preoperative planning right before RRBSO.

#### Survey of HRSCs in Israel:

Seven clinics in Israel perform biannual pelvic US and CA-125 starting at age 25-30 and one clinic from age 35 until RRBSO. One clinic currently performs pelvic US only per women’s request. Following RRBSO, four clinics do not recommend OvCa surveillance, while five continue annual/biannual US and CA-125. 

#### Final Recommendation:

Consultation with a gynecologist/gyneco-oncologist at age 30–35 for discussing surgical plan. Biannual pelvic US and CA-125 are recommended from age 35 until RRBSO. Pelvic US should be provided in tertiary centers by experienced sonographers, and women should be counseled regarding screening limitations. In cases of FH of OvCa before age 40, surveillance should be considered 5–10 years prior to age of familial OvCa diagnosis.

3. ***Surveillance for other malignancies***.

Surveillance for malignancies other than BC and OvCa represents a controversial area with evolving recommendations, as both risk evaluation and surveillance efficacy remain inadequately established. Since risk assessments for almost all cancer types exhibit substantial variability between studies [68], we focused discussion on malignancies that were consistently found to have a significantly higher risk among carriers [69]. Particular consideration was directed toward gastrointestinal neoplasms, encompassing pancreatic, gastric, biliary, and colorectal malignancies. The quantification of risk profiles and validation of screening modality efficacy for these malignancies remain unclear. Any consideration of surveillance should be discussed by a multidisciplinary team to evaluate surveillance effectiveness, false positive/uncertain result rates and impact on staging and survival outcomes. The Israel Gastroenterology & Liver Disease Association (IGA) is in the process of creating guidelines that will address these issues. The IGA currently recommends considering screening all *BRCA* PVCs for gastric and colorectal cancer, based on efficacy reports. However, as evidence is limited thus far, we recommend consultation with a gastroenterologist for all *BRCA* PVCs at age 50 or earlier if there is FH (first/second degree from same parental lineage as *BRCA*-PV), for risk assessment of pancreatic, gastric and colorectal cancer, and planning surveillance accordingly. Additional recommendations for each cancer type are listed below.

### **Pancreatic cancer (PanCa)**:

Background: PanCa is a rare but lethal cancer. Lifetime incidence in the general population is approximately 1.6% and 5-year survival is 10% [70]. Diagnosis at earlier stages of disease is associated with improved survival, with 93% 10-year survival for stage 0 and 35% 5-year survival for stage I cancers [71].

Among *BRCA1* and *BRCA2* carriers, lifetime PanCa relative risks (RR) are 2.36 and 3.34 respectively; sex-stratified RRs are as follows: male *BRCA1* 1.92[95% confidence interval(CI):1.12–3.28], female *BRCA1* 4.27(CI:2.01–9.05), male *BRCA2* 2.96(CI:1.78–4.94) and female *BRCA2* 4.34(CI:2.19–8.62) [69] However, absolute risks are low: male *BRCA1* 2.9% (CI 1.9–4.5%), female *BRCA1* 2.3% (CI 1.5–3.3%) male *BRCA2* 3% (CI 1.7–5.4%) and female *BRCA2* 2.3% (1.3–4.2%) [74]. Despite the critical need for early detection, screening efficacy remains questionable. Clinical trials have not demonstrated improved survival among screen-detected PanCa compared with controls [72]. Screening appears to be associated with stage shift and better 3-year survival than expected [72], but may involve false positives and unnecessary surgical interventions [73].

A retrospective multicenter cohort study of 180 asymptomatic Israeli BRCA1/2 carriers undergoing PanCa surveillance detected adenocarcinoma in 4 (1.6%) and grade 1 neuroendocrine tumor (G1-NET) in 4 others. All cancer cases occurred in *BRCA2* carriers and two had no FH of PanCa. Three patients presented with resectable disease, while one had a stage IIIB tumor. Among G1‐NET cases, one underwent surgical resection while others were managed with surveillance [71].

#### *International guidelines*:

ESMO guidelines recommend considering PanCa screening for *BRCA-PVCs* with a FH of PanCa (first/second degree relative from the same side of family as the *BRCA-*PV) and states that screening should be conducted in high volume centers ideally within clinical trial framework, following thorough discussion of potential limitations including cost, incidence of benign/indeterminate findings, and uncertainty regarding benefit.

Updated NCCN guidelines recommend considering screening for *BRCA1*-PVCs with FH, and all *BRCA2* carriers regardless of FH, preferably within a longitudinal study. Screening can be done using contrast-enhanced MRI/magnetic resonance cholangiopancreatography (MRCP) and/or endoscopic ultrasound (EUS).

The American Society for Gastrointestinal Endoscopy (ASGE) indicated a conditional recommendation for screening all *BRCA1/2* carriers, regardless of FH [70] and positions EUS as the preferred modality screening.

##### HRSCs in Israel:

four clinics recommend PanCa screening for BRCA-PVCs with a FH starting at age 50, while two recommend screening for all BRCA2 carriers over 50 years, regardless of FH. Three clinics do not have PanCa screening recommendations. 

#### Final Recommendation:

* BRCA1*-PVC: PanCa screening is recommended for those with FH from age 50 or 10 years earlier than PanCa diagnosis age in family. PanCa screening should be considered for all *BRCA2*-PVCs from age 50 regardless of FH, or 10 years earlier than PanCa diagnosis age in family for those with FH. As the suggested recommendation for all *BRCA2*-PVCs is very recent, centers will gradually update protocols accordingly.

### **Gastric Cancer**

*Background: *The risk of gastric cancer (GCa) is increased in BRCA-PVCs (BRCA1 RR = 2.17, BRCA2 RR = 3.69), with an absolute lifetime risk up to 3.5% in BRCA2 carriers [69]. Evidence exists for non-genetic modifiers, especially H. Pylori infection in countries with high GCa [74], for whom endoscopic screening efficacy has been demonstrated. A large meta-analysis reported that endoscopic screening was associated with a significant 42% reduction in GCa mortality [75].

#### International guidelines:

NCCN and ESMO guidelines do not include specific recommendations regarding GCa surveillance for BRCA-PVCs. The ACG (American college of Gastroenterology) recommends individualizing surveillance by genetic counseling and patient preference [76]. The AGA (American Gastroenterology Association) recommends considering gastroscopy and H pylori testing at age 45.

#### Survey of HRSCs in Israel:

three centers recommend GCa screening from age 50 if FH exists, while one recommends screening every 5 years starting age 45, regardless of FH. Five clinics do not have GCa screening recommendations.

#### Final Recommendation: BRCA-

PVCs with GCa FH should be referred to a gastro-genetic clinic for individual risk assessment and consideration of surveillance.

### **Gallbladder Cancer**

Gallbladder cancer risk is significantly increased for *BRCA1* carriers (RR = 3.34, *p* = 0.01), but the absolute risk is low (1.6%) [69]. Furthermore, no study has examined screening efficacy in *BRCA-*PVCs. Therefore, no surveillance recommendations exist in international guidelines or HRSC in Israel, and we do not recommend screening for gallbladder cancer.

### **Colorectal cancer (CRC)**

*Background*: CRC risk in *BRCA*-PVCs largely depends on FH [69, 76]

#### International guidelines

NCCN and ESMO guidelines do not include recommendations regarding CRC screening for BRCA-PV carriers.

#### HRSCs in Israel

three centers recommend screening for CRC according to FH, while one recommends screening every 5 years starting age 45, regardless of FH. The remaining reported no recommendations.

#### Final recommendation

For carriers with CRC FH, surveillance strategies should be guided individually following gastro-genetic consultation, usually starting at age 40, or 10 years younger than the youngest affected relative.

### Malignant Melanoma

Limited data suggest that *BRCA2* carriers may have a slightly higher risk of cutaneous malignant melanoma (MM) (RR = 2.58) [69, 77]. Only one study found a significant increase in ocular melanoma risk [78]. There is no evidence for effectiveness of surveillance by full-body skin examination.

#### International guidelines

The NCCN guidelines state that general melanoma risk management is appropriate, such as annual full-body skin examination and minimizing ultraviolet exposure.

#### Survey of HRSCs in Israel

three clinics recommend screening based on FH, two recommend annual surveillance, and four clinics do not recommend specific MM screening.

Final Recommendation: Minimize ultraviolet (UV) exposure.

## Discussion

Thirty years after the discovery of BRCA1/2, prevention and surveillance recommendations remain inconsistent across international guidelines (e.g. NCCN and ESMO), in part reflecting technological advances (e.g. breast MRI interpretation) and in part reflecting areas of uncertain evidence. This inconsistency is a source of confusion for both clinicians and women.

A recently published comprehensive review on national and international guidelines [16]ESMO guidelines with NCCN guidelines, and found several inconsistencies (e.g. recommendation for biannual breast MRI for *BRCA1* PVCs aged 25–40 vs. annual MRI and MG by ESMO and NCCN, respectively), This may drive institution-specific protocols over national guidelines. The authors advocated for unified protocols across hospital and community settings through expert consensus to resolve controversies. In our study, nine HRSCs, serving most BRCA1/2 carriers throughout the country, collaborated to compare and harmonize recommendations. We established a framework for national Israeli guidelines through a collaborative, multidisciplinary process involving specialists from the Israeli Consortium for HBOC to resolve disagreement via expert discussion and updated literature review.

The study identified areas of controversy in *BRCA*-PVC management. Breast imaging discrepancies included initiation age, intervals, modalities, and recommendations during pregnancy and breastfeeding. Our current recommendations include annual MRI from age 25, and addition of MG at six-month intervals for ages 30–75 (MRI can be considered an alternative to MG until age 40). Further research is needed to assess biannual MRI cost-effectiveness, particularly for *BRCA1*. During breastfeeding, protective benefits alongside imaging limitations should be discussed; MG ≥ 30 years should be considered, and breast US and MRI are currently recommended despite limited evidence, requiring further research.

Israeli HRSCs lack clarity regarding hysterectomy discussion before RRBSO. Additionally, most HRSCs continue biannual CA-125 and pelvic US following RRBSO despite lack of evidence and international guideline endorsement. We recommend OvCa surveillance exclusively for women aged 35 or older until RRBSO. Since OvCa surveillance was the common practice for many years, cessation requires adaptation of caregivers, and reassurance of its safety for women.

An important finding was that approximately half of HRSCs did not provide screening guidance for non-breast and ovarian malignancies, with inconsistencies among those providing it. Due to ongoing updates, we recommend consultation with a gastroenterologist for all *BRCA* PVCs at age 50 or 10 years prior to the earliest age of GI cancer diagnosis in the family, for risk assessment of pancreatic, gastric and colorectal cancer, and surveillance accordingly.

We recommend considering PanCa screening for all *BRCA2* carriers, and *BRCA1* carriers with PanCa FH, by EUS or MRI\MRCP, from age 50 or 10 years earlier than familial diagnosis age. Surveillance strategies for carriers with FH of CRC or GC, should be guided individually, following gastro-genetic counseling.

Our findings and recommendations have significant implications for Israeli healthcare policy, by optimizing resource allocation, eliminating unnecessary tests, and improving treatment options. For instance, current OvCa surveillance at younger ages and following RRBSO lacks evidence-based support, suggesting these resources should be redirected to other HRSC needs.

In Israel, the implementation of national *BRCA* PS has significantly increased numbers of identified carriers. This emphasizes the importance of consensus recommendations as a health policy goal, and accentuates the need for equitable access. Currently, HRSC clinics are mainly located in the Jerusalem and Tel-Aviv areas. This geographic disparity underscores the need for more balanced resource distribution. A national protocol is crucial for community-based healthcare delivery, where standardization faces unique challenges, especially in primary care settings. This standardization has significant implications for clinical practice, ensuring equitable, high-quality care delivery regardless of geographic location or healthcare setting. Finally, reimbursement policies for *BRCA*-PVCs warrant revision to incorporate recent surveillance recommendations. This includes expanding coverage for performing breast MRI every 6 months in BRCA1 carriers given its efficacy for early detection [21, 24]. Additionally, the recent suggestion of PanCa screening for all *BRCA2* carriers and *BRCA1* with FH requires HRSC adaptation ensuring access to gastroenterology consultation and availability of new screening modalities.

### Strengths and limitations

The main strength of this study is that our recommendations are based on current evidence coupled with multi-center and multidisciplinary consensus. The survey was of the nine leading Israeli HRSCs, country-wide, and we included leading physicians in all fields relevant to management of *BRCA*-PVCs. After multiple meetings we formed recommendations acceptable to all participants. We created an updated, comprehensive and practical protocol for managing unaffected *BRCA* PVCs.

The main limitation of the study is that although many of our recommendations are based published evidence, many recommendations remain based on expert consensus, as evidence was of low level or currently non-existent. This limitation is also true for other published guidelines. Another limitation is that our findings may not directly apply to other countries due to healthcare system differences, the scale of identified BRCA-PVCs, population composition and other factors. Moreover, cancer risks for *BRCA-PVCs* may be PV specific [79]and differ in ethnically diverse populations, where the spectrum of *BRCA*-PVs differs from AJ or Ethiopian founder PVs.

### Future directions

Our study indicates important gaps that should be addressed by further research, both generally for all *BRCA*-PVCs and specifically for the Israeli health system:

*The need for evidence for specific risk reduction and surveillance measures*. For breast cancer this includes breast imaging during pregnancy and lactation. For ovarian cancer, the efficacy of early salpingectomy and delayed oophorectomy. For non-breast/ovarian cancer, examining the efficacy of current measures, especially screening for pancreatic cancer, is critical.

*Personalized risk assessment* – Currently, risk assessment in *BRCA*-PVCs is usually not refined beyond consideration of the specific gene (*BRCA1* vs. *BRCA2*) and FH of various cancers. Achieving better refinement is important to tailoring both risk reduction and surveillance methods, including age of implementation. Models that also integrate polygenic risk scores (PRS) along with BRCA PVs, FH, and other clinical factors, could be helpful to assess risks more individually for each BRCA PVCs, and refine management recommendations accordingly. While not currently recommended for clinical use, several studies show that PRS can predict BC risk [80, 81], and models like BOADICEA [82] [implemented in the CanRisk tool [83] and Tyrer-Cuzick [84] have incorporated PRS into cancer risk calculation for *BRCA*-PVCs. Artificial intelligence (AI)-based cancer risk prediction models for BRCA-PVCs have recently been shown to improve discrimination over traditional models [85], especially when integrating clinical and genetic data with imaging. However, use of AI in the real-world setting as a guide for management will depend on validation, regulatory alignment, and integration into the workflow.

*Cell free DNA* (cfDNA) is an additional tool which has potential for noninvasive early detection of cancer, particularly in high-risk populations such as *BRCA*-PVCs [86]. Currently, cfDNA testing is primarily used in research settings and is not part of guideline-based screening [87].

#### Implementation research of the population screening program in Israel

PS is a new paradigm in detecting and managing women *BRCA*-PVCs, and implementation research is important in order to improve the current process. This includes assessment of awareness and uptake, and delivery of positive results. Outcomes research is important to determine rates of HRSC attendance and adherence to recommendations by *BRCA*-PVCs, as well as the effects of negative test results on health behavior (e.g. MG screening) in non-carriers. Long-term follow-up is necessary to assess the effect of PS on morbidity and mortality.

#### Expansion of the PS program

PS is currently based on testing for ancestry-specific *BRCA*-PVs in AJs and has recently been expanded to include *BRCA2*-PVs in Ethiopian Jews. Future development of PS could include expanding the test to full sequencing of *BRCA1/2* and perhaps other high-penetrance genes as well as including additional populations. This would require careful planning of how PVs would be detected and reported, and how management of PVCs would be handled. Extension of the current process is also likely to be challenging on a population-wide scale.

## Conclusions

Screening protocol harmonization at a national level is essential for optimal cancer risk reduction in high-risk women. When evidence-based guidelines are unavailable, expert consensus provides an adaptable framework that can incorporate emerging scientific evidence into healthcare policy. Data sharing throughout Israel with experts in multiple fields, and ongoing collaboration are important for optimal, up-to-date management of BRCA PVCs. As the *BRCA* carrier population expands, standardized protocols across hospital and community settings become increasingly critical for optimizing care, with the ultimate goal of reducing cancer morbidity and mortality.

**Table 1:** Summary of surveillance recommendations in high-risk surveillance clinics in Israel (the nine medical centers are listed in alphabetized order)

	Assaf Harofeh- Shamir	Assuta Ashdod	Belinson- Rabin	Hadassah (Mt. Scopus)		Ichilov	Kaplan	Rambam	Shaare Zedek	Sheba
SURVEILLANCE Breast Cancer Imaging				BRCA1	BRCA12					
Age 25–30 Once a year	MRI	MRI	MRI	MRI		MRI + CBE	MRI	MRI + CBE	MRI	MRI
Once a year	Breast US + CBE (± MG)	none	Breast US	CBE	CBE	MG (± US) + CBE	Breast US	Breast US + CBE	BRCA1 MG + CBE	Breast US
Age 30–35 Once a year	MRI	MRI	MRI	MRI	MRI	MRI + CBE	MRI	MRI + CBE	MRI	MRI
Once a year	MG ± Breast US + CBE	MG + Breast US	Breast US	MRI		MG ± breast US + CBE	MG + Breast US	MG Tomo + CBE	MG + Breast US + CBE	MG MLO
Age 35–75 Once a year	MRI	MRI till 70	MRI	MRI	MRI	MRI + CBE	MRI	MRI + CBE	MRI	MRI
Once a year	MG ± Breast US + CBE	MG + Breast US	35–40 Breast US > 40 MG + Breast US	MRI + 40y MG-MLO	MG q2y	MG (± US) + CBE	MG + Breast US	MG-tomo + CBE	MG + Breast US + CBE	MG 35-40y- MG MLO
Additional routine recommendations	> 75 same if well			> 75: MRIq2y, MGq2y				> 75 same if well	> 75 same if well	Up to 80y- same
Post RRM- residual tissue Once a year	MRI	NA	Breast US or MRI	CBE q6M + MRI		CBE q6M + MRI	Breast US or MRI	MRI q2y + CBE	MRI + CBE	MRI q2y, USq2y
Post RRM- minimal residual tissue	Breast US q2y MRI q2y CBE q1y	NA	NA	CBE q6M		CBE q6M, MRI q2y	Breast US q1y	Breast US q2y + CBE q1y	Breast US + CBE q1y	Breast US q2y
Pregnancy and lactation	Breast US + CBE q6M, MRI 2 M after lactation	Breast US q6M	Breast US q3m, MRI 6 M after lactation	Breast US + CBE q6M		Pregnancy: CBE q3m Lactation: Breast US/MG q3m	NA	Breast US q3m + Breast CBE + MRI 6 M after lactation.	Breast US + CBE q6M, MRI 2 M after lactation	Pregnancy: Breast US q3M, MG, MRI 3 M after delivery. MRI 2 M after lactation.
SURVEILLANCE Ovarian cancer										
Age 30–35 (Biannual)	CA125 + Pelvic US (Annual)	CA125 + Pelvic US	CA125 + Pelvic US	CA125 + Pelvic US		CA125 + Pelvic US	CA125 + Pelvic US	CA125 + Pelvic US	none	none*
35y-RRBSO (Biannual)	CA125 + Pelvic US (Annual)	CA125 + Pelvic US	CA125 + Pelvic US	CA125 + Pelvic US		CA125 + Pelvic US	CA125 + Pelvic US	CA125 + Pelvic US	CA125 + Pelvic US	none*
Following RRBSO (biannual)	CA125 + Pelvic US (q1y)	none	CA125 + Pelvic US (q1y)	none		CA125 + Pelvic US, offer reducing frequency	CA125 + Pelvic US	CA125 + Pelvic US	none	none
SURVEILLANCE Other Malignancy										
Pancreatic Cancer**	FH based	> 50 FH based		None		BRCA1: FH based BRCA2: >50	None	BRCA1: FH based BRCA2: >50	FH based > 50 or 10 yrs before age of family member diagnosed with pancreatic	FH based > 50 or 10 yrs before age of family member diagnosed with pancreatic
GI	none	> 50 FH based		none			None	> 45 q5y Gastroscopy + Colonoscopy	FH based Gastroscopy +/or Colonoscopy	FH based Gastroscopy +/or Colonoscopy
									Colonoscopy: FH based	Colonoscopy: FH based
Melanoma	q1y (skin)	> 50 FH based		none		FH based	None	q1y (skin, eye)	FH based	none

*pelvic US is performed by women’s request due to previous guidelines including US

**Currently undergoing update as new NCCN guidelines (version 1.2026) recently emerged with screening recommendation consideration for all *BRCA2* > 50

CBE- clinical breast examination; FH- family history; MRI -magnetic resonance imaging; MG- Mammography, US- Ultrasound, PE- physical examination; MLO- mediolateral oblique; OCP- Oral contraception pill; HRT- hormone replacement therapy; RRM- Risk reducing mastectomy; RRBSO- Risk reducing Bilateral Salpingo-oophorectomy. NA = no answer

**Table 2:** Summary of recommendations of the Israeli Consortium, NCCN and ESMO for surveillance and risk-reduction for unaffected *BRCA1/2* PV carriers

			Israeli consortium	NCCN	ESMO
Breast Cancer					
Surveillance	**Age at initiation**		≥ 25 years or individualized based on FH of BC < age 30	≥ 25 years or individualized based on FH of BC < age 30	≥ 30 years or 5 years before youngest family member age at BC diagnosis
	**Imaging Modality**	**25–29**	Annual CBE [C] Annual MRI [B] *BRCA1* PV: consider interval MG, US or additional MRI between annual imaging [C].	CBE 6 monthly Annual MRI or MG if MRI unavailable.	No measures recommended.
		**30–75**	Annual CBE [C] Annual MRI (A). MG at 6-month intervals. Until age 40, US can be considered instead of MG [C]	CBE 6 monthly Annual MRI and MG	***BRCA1*-** Biannual MRI [A] If MRI is unavailable: Age 30–40- US; ≥40- MG [C] ***BRCA2****-* Annual MRI [A]
		**> 75**	Individualized [C]	Individualized	Individualized
	**Pregnancy and Lactation**		Annual CBE [C] Breast US every 3–6 m [C] MRI 2 m after stopping breastfeeding; avoid pregnancy before performing MRI. [C]	No recommendation noted	No recommendation noted
	**Following RR-M**		Post-operative MRI 6–12 m after surgery to assess residual breast tissue[C]. **No significant residual breast tissue**: annual CBE ± US [C]. **Significant residual tissue**: individualized; consider annual CBE + annual MRI or consider repeat surgery [C].	CBE	Post-operative MRI 6–12 months after surgery to assess residual breast tissue. Individualized follow up [C].
Pharmacologic prevention			Discuss risks and benefits of SERM with *BRCA2* PVCs not planning RRM [C].	Consider selective ER modulators, including discussion of risks and benefits.	Consider SERM modulators [C].
Risk-Reducing Surgery	**Age**		RRM should be discussed with individual tailored decision-making [B], ideally 30–55.	RRM should be discussed with individual tailored decision making.	RRM should be discussed with individual tailored decision-making [B].
	**Preoperative imaging**		Breast MRI within 2 months prior to surgery [C]	No recommendation noted.	No recommendation noted.
	**Type of surgery**		Immediate reconstruction should be offered [C].	No recommendation noted	Immediate reconstruction should be offered [C].
Ovarian Cancer					
Surveillance	**Age**	**≤ 35**	No routine surveillance. In families with OC diagnosed ≤ 40, surveillance should begin 10 years prior to the age of OC patient [C].	No recommendation noted. Consultation with gynecologic oncologist/or gynecologist with expertise in genetic susceptibility to gynecologic cancer.	No recommendation noted.
		**35 Until RR BSO**:	Bi-annual pelvic US and CA-125 testing, with counselling on the limitations of these tests[C].	Preoperative CA-125 and Pelvic US	Bi-annual pelvic US and CA-125 testing, with counselling on the limitations of these tests[C].
	**Patients declining Surgery**:		Continued annual US and CA-125 testing, with counselling on the limitations of these tests[C].	No recommendation noted.	No recommendation noted.
	**Following RRSO**		No screening [D].		No screening [D].
Risk-Reducing Surgery	**Age**		***BRCA1***: ages 35–40. ***BRCA2***: ages 40–45 [A]	***BRCA1***: *35–40 years.* ***BRCA2***: *40–45* unless age at OC diagnosis in the family suggests earlier age for surgery.	***BRCA1***: ages 35–40. ***BRCA2***: ages 40–45 [A].
	**Preoperative planning**		Pelvic US and Ca125. Address bone, cardiovascular, neurologic psychosocial and sexual health, and quality-of-life aspects of RRSO. Preoperative menopause management consultation if patient premenopausal.	Pelvic US and Ca125. Address bone, cardiovascular, neurologic psychosocial and sexual health, and quality-of-life aspects of RRSO. Preoperative menopause management consultation if patient premenopausal.	No specific recommendation.
	**Type of surgery**	**RRBSO**	RRSO is the standard recommendation [A].	RRSO is the standard recommendation^4^.	RRSO is the standard recommendation (A).
		**RRS**	RRS is not recommended outside clinical trial setting[C].	RRS is an option for premenopausal patients not ready for oophorectomy. Completion oophorectomy is recommended as per guidelines, unless specified by clinical trial protocol. Strong consideration of participation in a clinical trial.	RRS is not recommended outside clinical trial setting [C].
		**Hysterectomy**	Discuss with individualized recommendation based on risk factors for uterine cancer, desire for estrogen-only HRT, surgical risks, and psychological factors [C].	Discuss the risks and benefits of concurrent hysterectomy at the time of RRSO prior to surgery. Individuals who undergo hysterectomy are candidates for estrogen-alone HRT.	No recommendation noted
Pancreatic cancer					
Surveillance		***BRCA1***: from age 50 (or 5–10 years younger than affected relative, whichever is earlier) **in carriers with ≥ 1 1st/2nd degree relative** with exocrine pancreatic cancer [C]. ***BRCA2***: consider for **all** carriers from age 50 (or 10 years younger than the earliest pancreatic cancer diagnosis in the family, whichever earlier) [C].		***BRCA1***: from age 50 (or 5–10 years younger than affected relative, whichever is earlier) **in carriers with a ≥ 1 1st/2nd degree relative** with exocrine pancreatic cancer ***BRCA2***: consider for **all** carriers from age 50 (or 10 years younger than the earliest pancreatic cancer diagnosis in the family, whichever earlier).	***BRCA1***,***2***: Consider from age 50 (or 5–10 years younger than affected relative) **in carriers with at ≥ 1 1st/2nd degree relative** with exocrine pancreatic cancer
Screening modality (annual)		EUS or contrast-enhanced MRI [C].		Contrast-enhanced MRI/MRCP and/or EUS.	Contrast-enhanced MRI and/or EUS [C]. Screen in center with high volume experience [C] and as part of a clinical trial [A].
Gastric and Colorectal Cancer					
		Refer individuals with FH of gastric/colorectal cancer to a genetic clinic specializing in gastrointestinal cancers for individual risk management, at age 50 or 10 years before the youngest relative’s diagnosis age [C].		No recommendation noted	No recommendation noted.
Gynecological, Endocrinological issues and General Health recommendations					
Fertility preservation		Should be discussed with women without children from age 30. [B]		Refer to fertility specialist if desired for discussion of in vitro fertilization, oocyte/embryo-cryopreservation.	Encourage to complete childbearing before recommended RRBSO age [A]; if not feasible, offer oocyte/embryo cryopreservation[B].
PGT		Discuss PGT option [A]. Refer to a fertility specialist if desired.		Consider PGT, gestational carrier, and adoption.	Inform about PGT option [A].
Oral contraception		Consider as contraception, but not for OC risk-reduction (d/t to slight increase in BC risk).		Consider combination estrogen & progestin contraception for ovulation suppression.	Decisions about hormonal contraception should weigh possible increase in BC against contraceptive efficacy, convenience and OC risk reduction [C].
HRT Age and Type of HRT		**If RRSO ≤ Age 45**: Recommended for individuals without contraindication (ex. BC)until age 50; reassessment thereafter. [C] **If RRSO performed ≥ Age 45**: individualized [C] Combined HRT if uterus intact; estrogen-only after hysterectomy. Topical vaginal estrogen recommended for treating vaginal atrophy [C].		Consider for premenopausal women without contraindications (such as BC). If uterus is intact, consider LNG-IUD with oral/transdermal estrogen. Combined HRT with counselling regarding bleeding and endometrial cancer risk/awareness. Combination estrogen with SERM. Combination OCPs that can be taken continuously.	Consider for menopausal symptoms after discussing limitations and risks [C]. Low-dose intravaginal estrogens may be considered to manage genitourinary symptoms of menopause [C].
Bone density		Baseline bone density scan recommended 6 months post-RRSO. **Follow-up scan- Without HRT**: Annually; **With HRT**: every 3 years[C].		No specific recommendation noted.	Consider bone assessment, tailor to individual risk factors. Consider preventive/therapeutic measures as indicated [B].
Lifestyle and general health		Encourage regular physical activity and maintaining normal BMI [B]. Minimize alcohol intake [C]. Minimize UV exposure. Avoid smoking. Continue routine gynecological screening (such as for cervical cancer).		Annual full-body skin examination and minimizing UV exposure.	Encourage physical exercise if appropriate. Avoid being overweight or obese and encourage breastfeeding [B]. Minimize alcohol intake [C].
Psychological health		Offer psychological counselling and/or further support [B]			Offer referrals for psychological counselling and/or further support [B]
Sexual health		Offer sexual health support regardless of age, partner status or sexual orientation [A].			Sexual health concerns should be assessed regardless of age with support and resources offered as needed [A].

BC- Breast cancer; BMI- Body mass index; Ca125- Cancer antigen 125; CBE- Clinical breast examination; EUS- endoscopic ultrasound; FH- family history; HRSC- High risk surveillance clinic; HRT- hormone replacement therapy; LNG-IUD- levonorgestrel-releasing intrauterine device; m-month; MRI -magnetic resonance imaging; MG- Mammography, MRCP- magnetic resonance cholangiopancreatography; OC- Ovarian cancer; PGT- Pregestational testing; RRBSO- Risk reducing Bilateral Salpingo-oophorectomy; RRM- Risk reducing mastectomy; RRS- Risk reducing salpingectomy; SERM- selective estrogen receptor modulator; US- Ultrasound; UV- ultraviolet

^1^Level of evidence based on ESMO Clinical Practice Guidelines 2023 (see supplement 2)^88,89^.

^2^All recommendations are category 2 A = “Based upon lower-level evidence, there is uniform NCCN consensus (> 85% support of the Panel) that the intervention is appropriate” (NCCN Guidelines Version 1.2026Genetic/Familial High-Risk Assessment: Breast, Ovarian, Pancreatic, and Prostate)

^3^Currently, no unequivocal recommendation exists for MRI twice a year due to insufficient evidence, however, biannual MRI for BRCA1 carriers, 25-40y) with the addition of MG every 2 years can be offered in centers where available. In BRCA carriers that cannot perform breast MRI, MG should be considered every 2 years. For carriers undergoing the above outlined surveillance, routine breast US is unnecessary

^4^Discuss RRM including degree of protection, reconstruction options, risks, FH and residual breast cancer risk with age and life expectancy. Psychosocial and quality-of-life aspects of undergoing RRM should be addressed

^5^Principles of Surgery: Appropriate surgical and pathologic expertise is strongly recommended. Sectioning and Extensively Examining the Fimbriated End (SEE-FIM) protocol for pathologic assessment and pelvic washings should be performed If serous tubal intraepithelial carcinoma (STIC lesion) is found, further consultation with a gynecologist oncologist is recommended

^6^For individuals considering pancreatic cancer screening this should be performed in experienced high-volume centers. following an in-depth discussion about the potential limitations to screening, including cost, the high incidence of benign or indeterminate pancreatic abnormalities, and uncertainties about the potential benefits of pancreatic cancer screening

^7^from same parental lineage as PV

^8^In women with a history of BC: HRT is usually contraindicated, however, in triple negative BC HRT can be considered by oncology consultation

## Supplementary Information

Supplementary Material 1

Supplementary Material 2

## Data Availability

Not applicable.
