# Supplementary material for: Managing healthcare for female BRCA carriers in the population screening era: developing a harmonized national policy for surveillance and risk-reduction
Source: Isr J Health Policy Res. 2026 Jan 30;15:4. doi: 10.1186/s13584-026-00746-3 (PMC12857140; doi:10.1186/s13584-026-00746-3)
Supplement: Supplementary file 1 — Supplementary Material 1 [file 13584_2026_746_MOESM1_ESM.docx]

**Recommendations for surveillance and risk-reducing strategies for BRCA PV carriers**

These recommendations are based on the combined protocol developed at the nine leading high-risk surveillance clinics (HRSC) in Israel, through comprehensive meetings of healthcare specialists discussing surveillance and risk reducing recommendation in female BRCA carriers.

The expert panel included specialists from multiple fields: medical genetics, genetic counseling, radiology, breast surgery, plastic and reconstructive surgery, medical oncology, gynecology, gynecologic oncology, menopause medicine and gastroenterology.

The recommendations incorporate established international standards, including those from the National Comprehensive Cancer Network (NCCN) and European Society for Medical Oncology (ESMO), while also addressing controversial areas through clinical consensus discussions. The differences between Israeli and NCCN/ESMO guidelines are noted. When not otherwise noted, our recommendations are the same as for NCCN/ESMO.

1. **Breast cancer**
2. **Ovarian cancer**
3. **Gynecology and Endocrinology related issues**
4. **Other cancers**
5. **Lifestyle**
6. **Breast cancer**
7. **Breast Cancer** **Risk-reduction in Healthy BRCA Carriers:**

- **Risk reducing surgery**

Routine consultation with a breast surgeon regarding risk reducing mastectomy for all BRCA carriers should be conducted during the first clinic visit. Subsequent consultations are recommended after a year and then every 6 months to three years until age 50.

**Discussion should include the following points:**

1. Risk reducing bilateral mastectomy (RRM) reduces the risk of developing cancer by at least 90% (1). For women carrying the BRCA1 PV, there is a survival advantage for RRM, while in BRCA2 carriers, the trend was similar but not statistically significant. The greatest benefit is seen between the ages of 30-55(2).
2. Additional benefits of RRM compared to surveillance and early detection of BC include:

- Most BRCA1 carriers (~80%) and about half of BRCA2 carriers, if diagnosed with BC, will likely undergo chemotherapy. This is due to the high rate of triple-negative breast cancer (TNBC) in BRCA1 carriers or high-risk genomic profiles even in luminal tumors (including BRCA2 carriers)(3). Endocrine therapy is also commonly required for luminal tumors. Based on our estimate, given the characteristics of expected tumors, 80-90% of BRCA1 carriers will receive chemotherapy, and about 50% of BRCA2 carriers.
- Additional treatment consequences after cancer diagnosis: need for endocrine therapy in cases of hormone receptor-positive tumors (about 70-75% of BRCA2 carriers and 40% of BRCA1 carriers). In some cases, radiotherapy is indicated (even after mastectomy), which may also increase long-term morbidity and affect aesthetic outcomes (4,5).
- Surgical extent after BC diagnosis and implications:

The risk of lymphedema after treatment for breast cancer is estimated at 1.7-5% (6,7). Aesthetic outcomes are often better after risk reducing surgery compared to surgery after disease diagnosis, due to higher rates of nipple-areolar complex preservation, availability of more reconstruction options and scars that results in more symmetric and aesthetic outcomes(4,5).

**Timing of Surgery**:

The greatest health benefit from RRM is noted when surgery is performed between ages 30-55. Breast cancer risk is low at younger ages (age stratified risk tables by age are included at the end of the document(8)**.**

**Type of Surgery**:

- RRM does not need to include sentinel lymph node removal.
- For women wishing immediate reconstruction, Nipple-sparing mastectomy, when anatomically feasible, has sensory and aesthetic advantages. Preservation of the nipple areolar complex does not increase risk of disease. RRM without reconstruction- aesthetic flat closure- is an option for those who did not wish to have reconstruction.
- Preoperative breast MRI should be performed within 2 months prior to surgery.
- For risk reducing mastectomy with immediate reconstruction, a post-operative complication rate of about 15% can be expected. Complications may include flap ischemia & necrosis, infection, bleeding seroma. Loss of implants due to post-operative complications may occur in up to 5% of cases. Loss of sensation in the reconstructed breast occurs in almost all cases and persistent paresthesia or pain is possible (9).
- **Pharmacological Prevention:**

Tamoxifen improves survival of patients with estrogen receptor positive BC, including BRCA2 PVCs (10). Regarding cancer-free BRCA2 PVCs, although tamoxifen reduces BC risk, there is limited data available as to survival benefit, thus no conclusion can be drawn. Tamoxifen should be discussed with BRCA2 PVCs who choose not to undergo RRM, but not routinely recommended as pharmacologic prevention, especially as side effects can be severe.(10).

1. **Recommendations for Breast Cancer Surveillance in healthy BRCA Carriers:**

**Breast imaging:**

- **Ages 25-29**: Annual CBE and annual MRI. For *BRCA1* PV carriers, breast ultrasound (US) mammography (MG), or additional MRI at 6-month intervals can be considered.
- **Ages 30-75**: Annual CBE and annual MRI. Additionally, MG at 6-month intervals. Until age 40, breast US can be considered instead of MG.
  - Currently, no unequivocal recommendation exists for MRI twice a year due to insufficient evidence from small studies, however, bi-annual MRI for BRCA1 carriers (from age 25 to 40), with the addition of MG every 2 years, can be offered in centers where available.
  - For BRCA carriers that cannot perform breast MRI, contrast enhanced mammography (CME) should be considered every 2 years.
- **Post-75 years**: Follow-up schedules should be individualized based on woman’s health status. Healthy individuals may continue the same surveillance schedule as younger women.
- For carriers undergoing the above outlined surveillance, routine breast US is unnecessary.

**Surveillance During Pregnancy and Breastfeeding:**

Pregnancy and lactation present a challenge for breast cancer screening. The increased mammographic density and MRI background parenchyma enhancement impede test interpretation. *Imaging is performed by breast US at least every six months.*

- Pregnancy:
  - Dynamic contrast-enhanced MRI (DCE-MRI) of the breast is contraindicated during pregnancy due to concerns regarding fetal exposure to gadolinium.
  - Annual CBE
  - Breast US at least every six months.
- Breastfeeding:
  - Discuss breastfeeding duration due to limited imaging capabilities during lactation
  - Annual CBE
  - Breast US at least every six months. Mammography (focused on macrocalcifications) is recommended two months after delivery for women 30 years and above.
  - MRI is recommended two months after stopping breastfeeding; avoid pregnancy before performing MRI.

**Post-** RRM **Surveillance:**

- The annual breast cancer incidence following RRM is estimated as 0.08% and 0.21% in BRCA1 and BRCA2 respectively, which is lower than average risk women(11).
- **Follow-up:**
  - Post-operative MRI should be performed 6-12 months after surgery to assess residual breast tissue.
  - If no significant amount of breast tissue remains, annual clinical exam should be performed, with or without US, focusing on lymph nodes and residual breast tissue.
  - For significant residual tissue, consider repeat surgery or annual MRI.

1. **Secondary Breast Cancer Prevention in BRCA Carriers Diagnosed with Breast Cancer:**

**Contralateral Risk Reducing Mastectomy Post-Breast Cancer Diagnosis:**

In BRCA carriers, contralateral mastectomy after disease reduces mortality. The survival advantage for contralateral risk reducing surgery was greater in women under 40, those whose first tumor was poorly differentiated, non-triple-negative, and those who did not receive chemotherapy. Age and hormonal status influence the risk of developing cancer in the contralateral breast (12). Risk of contralateral BC increases over time, especially after five years, with a slightly higher incidence in BRCA1 compared to BRCA2 carriers (12,13).

- BRCA carriers diagnosed with breast cancer (including DCIS) should consult with a surgical oncologist or breast surgeon before surgery to discuss surgical options.
- For DCIS or STAGE I tumors- contralateral RRM (CRRM) may be recommended.
- Stage II tumors: For those requiring radiation and for patients with a higher risk of recurrence, the benefit of contralateral mastectomy is less clear. We recommend consulting with an oncologist and possibly re-assessing CRRM several years after the initial diagnosis. These cases should be discussed by a multidisciplinary team.

1. **Breast Cancer Surveillance in Breast Cancer Survivors:**

Annual risk for ipsilateral or contralateral BC is estimated at 2.5-5.5%, therefore surveillance should continue as recommended for healthy BRCA carriers(14,15).

- **Post-Lumpectomy**: annual surveillance with MRI, MG, and US as outlined for healthy carriers.
- **Post-RRM**:
  - An MRI should be performed at about six months post-surgery to assess residual tissue.
  - Further imaging depends on the radiologist's recommendation, with at least annual US. For women with advanced disease stage at diagnosis, MRI should be considered. Following discharge from oncologic follow up, surveillance should continue in the framework of a HRSC.

1. **Surveillance and Risk-Reduction for BC following Ovarian Cancer diagnosis:**

In recent years, with the advancement of maintenance therapy using PARP inhibitors, survival rates following ovarian cancer have significantly improved(16,17). Given these improved survival rates, attention to the risk of secondary malignancies, including breast cancer, has become essential. The risk of breast cancer after ovarian cancer in BRCA Carriers is approximately 6–14%, with an average risk of ~10% (18–20). The median time to breast cancer diagnosis after ovarian cancer is 50 months, and in the majority of cases BC will be diagnosed as early-stage disease.

**Follow up:**

- First 5 Years Post-Diagnosis: Monitoring is conducted by the treating oncologist.
- After 5 Years: BRCA carriers in remission from ovarian cancer may return to the HRSC for follow-up.

**Surveillance and Prevention**

**Stages I-II ovarian cancer**: Annual breast screening with MG and MRI.

**Stages III-IV** ovarian cancer: Annual MRI and clinical breast examination during the first five years post-diagnosis.

RRM should be individualized and may be considered 510 years after ovarian cancer diagnosis, in disease-free women <55 years of age.

**Ovarian Cancer Prevention and Surveillance**

- **Risk reducing surgery**

Routine consultation with a gynecologist/gyneco-oncologist regarding risk reducing surgery for all BRCA carriers should be conducted at age 30-35 years.

**I. Risk Reducing Surgery for Ovarian and Fallopian Tube Cancer:**

Risk-Reducing Salpingo-Oophorectomy (RRSO) has been shown to reduce the risk of ovarian cancer and all-cause mortality(21,22)

- **Age for Risk-Reducing Surgery:**
  - *BRCA1 Carriers*: Recommended between ages 35–40. The cumulative risk of ovarian cancer between ages 30–40 is 2% and between ages 40–50 is 8%(23).
  - *BRCA2 Carriers*: Recommended between ages 40–45. The risk of ovarian cancer between ages 40–50 is less than 1%(23).
  - In the rare case of premature menopause, immediate RRSO is advised, regardless of age.
- **Type of Surgery:**
  - Risk-Reducing Salpingo-Oophorectomy (RRSO) is the standard recommendation.
- **Salpingectomy Alone:**
  - Current evidence is insufficient to recommend preventive salpingectomy (with delayed oophorectomy) as clinical trials are ongoing.

For patients requesting salpingectomy or considering no surgery at all, the approach should include:

- **Risk Management Consultation:**
  - Engage a multidisciplinary discussion with the patient.
  - Highlight the limited data on the efficacy of salpingectomy alone.
- **Comprehensive Discussion:**
  - Address the lack of proven risk reduction with salpingectomy alone.
  - Emphasize the need for a second surgery, which carries its own risks.
  - Clarify that the recommended timing for oophorectomy remains unchanged, even if procedures are split.
- **Hysterectomy at the Time of RRSO:**
  - No general recommendation for routine hysterectomy, since the absolute risk is low (EC ~3.0%; USPC ~1.1%)(41) However, it should be discussed as a possibility for risk-reduction of uterine cancer, particularly in *BRCA1* carriers.
  - Hysterectomy may allow the use of estrogen-only hormone replacement therapy (HRT), which avoids the increased breast cancer risk associated with combined estrogen-progestin therapy.
  - This is particularly relevant for young *BRCA1* carriers planning long-term HRT or those with a history of breast cancer taking tamoxifen.
  - The decision to undergo hysterectomy should also consider surgical risks, prior surgeries, BMI, age, HRT use, and potential psychological impacts.

**II. Surveillance**

In the general population: large-scale studies have shown that surveillance including Ca125, US (US), and US+CA125 have shown downstaging, however this did not result in improved overall survival of ovarian cancer (24). For BRCA PV Carriers: a review of 6 international guidelines concluded that surveillance for ovarian cancer in BRCA PV carriers is not effective in reducing mortality(25).

- **Up to Age 35:** No routine ovarian cancer surveillance, except in families with ovarian cancer diagnosed before age 40, for whom surveillance should begin 10 years prior to the age of the youngest OC patient.
- **Ages 35 Until Preventive Surgery:** Bi-annual US (in tertiary centers by experienced sonographers) and CA-125 testing, with counselling on the limitations of these tests. US should be performed when planning for surgery

**For Patients Who Forego Preventive Surgery:**

- Continued surveillance with annual US and CA-125 testing**.**

**After RRSO:**

- Residual risk of peritoneal cancer (~1.5%), typically occurring 12–48 months post-surgery(26).
- No effective screening exists for peritoneal cancer; therefore, no routine ovarian cancer surveillance is recommended post-RRSO.
- *Patients with STIC (Serous Tubal Intraepithelial Carcinoma):* should consult a gynecologic oncologist.

1. **Gynecological and Endocrinological topics / issues**

**Reproductive considerations:**

**Fertility Preservation:**

Current evidence suggests a decrease in ovarian reserve in BRCA1 carriers, but not for BRCA2, but response to fertility treatment is not reduced(27–29). No data indicates increased breast cancer risk from hormonal fertility treatments in BRCA carriers(30,31) consistent with observations in non-carriers(32)**.**

Fertility preservation should be discussed with women without children starting at age 30, including women that are not in family partnership. Initial discussions on family planning should occur at the first clinic visit, with follow-up at age 30.

Pregestational testing (PGT):

PGT for BRCA PVs should be discussed with women at childbearing age, including risks and benefits of this technology. If desired, women should be referred to a fertility specialist for discussion.

**Contraception:**

- Oral contraceptives are not specifically recommended to reduce ovarian cancer risk due to a slight increase in breast cancer risk(33–35) . They remain a viable option for pregnancy prevention.
- In women with a history of breast cancer, oral contraceptives and hormonal IUDs (e.g., Mirena) are usually contraindicated. Non-hormonal IUDs or mechanical contraceptive methods are recommended**.**

**Hormone Replacement Therapy (HRT):**

- In the general population, there is increased morbidity and mortality in women who undergo RRSO before age 45, especially when performed before age 40, due to conditions like cardiovascular diseases, mood disorders, cognitive decline (memory and concentration problems), and bone density loss(36). Refer all women undergoing RRSO to counselling on psychological and sexual health.
- ***After RRSO Before Age 45:*** Routine HRT until age 50 for patients without contraindications with a reassessment thereafter.
- ***RRSO After Age 45:*** HRT decisions should be individualized.
- ***Choice of Therapy in Healthy BRCA carriers:*** Women under age 50 can choose HRT based on personal preference and side effects, as no specific formulations have shown a lower breast cancer risk in studies at time of writing these guidelines. Micronized progesterone or dehydrogesterone may be preferred. Topical vaginal estrogens are of critical importance for prevention and treatment of vaginal dryness.
- Combined estrogen-progestin therapy is necessary for women with an intact uterus, while estrogen-only therapy can be used after hysterectomy.
- In women with a history of BC, HRT is contraindicated, however, for triple negative BC it can be considered in an oncology consultation.

**Bone Density**

- A baseline bone density scan is recommended at six months post-RRSO.
- **Follow-up intervals depend on HRT use:**
  - **Without HRT:** Annual scans.
  - **With HRT:** Scans every three years.

1. **Other malignancies:**

Surveillance for malignancies other than BC and OC represents a controversial area with evolving recommendations, as both risk evaluation and surveillance efficacy remain inadequately established, and there is substantial variability between studies. Regarding gastrointestinal malignancies (pancreatic, gastric, biliary, and colorectal), quantification of risk profiles and validation of screening modality efficacy for these malignancies remain unclear.

- Since evidence is limited thus far, we recommend routine consultation with a gastroenterologist for all BRCA PVCs at age 50, for risk assessment of pancreatic, gastric and colorectal cancer, and surveillance accordingly.
  - 1. **Pancreatic cancer surveillance**

BRCA carriers are at increased risk for pancreatic cancer with a RR of 2.36 and 3.34 for BRCA1 and BRCA2, respectively(37).

- All BRCA2 carriers are recommended to consider pancreatic screening.
- BRCA1 carriers with a 1st or 2nd degree relative with pancreatic cancer (from the same parental lineage as the PV) are recommended to undergo screening.
- Pancreatic screening: endoscopic US or MRI, beginning at age 50 or 10 years earlier than age at pancreatic cancer diagnosis in the family.
  - 1. **Gastric Cancer Surveillance**

There is an increase in gastric cancer risk for BRCA1 and BRCA2 PV carriers. However, the absolute lifetime risk for BRCA2 PV carriers is only about 3.5% (37).

- - Women with a family history of gastric cancer should be referred to a genetic clinic specializing in gastrointestinal cancers for individual risk assessment and management planning, starting at age 50 or 10 years before the youngest relative’s age at diagnosis.
  - Further research is needed to establish the efficacy of endoscopy and/or H. Pylori testing as routine screening for all *BRCA* carriers.

**III. Gallbladder Cancer**:

The absolute risk for gallbladder cancer in BRCA PV carriers is low (e.g., 1.6% for BRCA1).

- No specific surveillance recommendations are made.

**IV. Colorectal Cancer**:

- - Women with a family history of colorectal cancer should be referred to a genetic clinic specializing in gastrointestinal cancers for individual risk assessment and management planning, starting at age 50 or 10 years before the youngest relative’s age at diagnosis.

**V. Melanoma**:

BRCA2 carriers may have a slightly higher risk of melanoma (RR = 2.5), though evidence is limited(39); no special recommendations exist for melanoma surveillance. Only one study found a significant increase in risk for Ocular Melanoma in BRCA PV carriers(40); no special recommendations exist for ocular melanoma surveillance.

- - An annual skin examination can be considered when there are family history of skin cancer or personal risk factors (i.e., fair skin or intensive sun exposure) exist.

1. **Lifestyle and Health Behavior Recommendations for BRCA PV Carriers:**

- Encourage regular physical activity and maintaining a healthy body weight in BRCA PV carriers, which may be even more important than in the general population(41).
- BRCA carriers should be counselled about the increased risk of breast cancer associated with alcohol consumption and be advised to limit alcohol intake to 1-2 drinks per week.
- Sunscreen use and avoiding smoking should be recommended.
- Emphasize that HRSC Clinic surveillance is not a substitute for routine gynecological screening (e.g., standard cervical cancer screen) which is recommended as for the general population.

__________________________________________________________________________________

Table 1: Cumulative incidence of breast and ovarian cancer among women with PVs in *BRCA1 or BRCA2* ascertained via unaffected AJ males (*Based on Gabai-Kapara et al, Table 1*(8)**)**.

|  | To Age, y | BRCA1 (SE) | BRCA2 (SE) |
| --- | --- | --- | --- |
| Risk of Breast Cancer | | | |
|  | 30 | 0.02 (0.02) | 0 |
|  | 40 | 0.17 (0.04) | 0.04 (0.03) |
|  | 50 | 0.35 (0.06) | 0.09 (0.05) |
|  | 60 | 0.41 (0.06) | 0.26 (0.08) |
|  | 70 | 0.52 (0.08) | 0.32 (0.09) |
|  | 80 | 0.60 (0.10) | 0.40 (0.11) |
| Risk of Ovarian cancer | | | |
|  | 40 | 0 | 0 |
|  | 50 | 0.05 (0.03) | 0.03 (0.03) |
|  | 60 | 0.27 (0.07) | 0.07 (0.05) |
|  | 70 | 0.47 (0.10) | 0.13 (0.07) |
|  | 80 | 0.53 (0.11) | 0.62 (0.18) |
| Risk of either Breast or Ovarian Cancer |  |  |  |
|  | 30 | 0.03 (0.02) | 0 |
|  | 40 | 0.23 (0.05) | 0.04 (0.03) |
|  | 50 | 0.41 (0.06) | 0.16 (0.06) |
|  | 60 | 0.60 (0.07) | 0.33 (0.09) |
|  | 70 | 0.77 (0.07) | 0.47 (0.11) |
|  | 80 | 0.83 (0.07) | 0.76 (0.13) |

**References**

1. Rebbeck TR, Friebel T, Lynch HT, Neuhausen SL, van ’t Veer L, Garber JE, et al. Bilateral prophylactic mastectomy reduces breast cancer risk in BRCA1 and BRCA2 mutation carriers: the PROSE Study Group. J Clin Oncol. 2004 Mar 15;22(6):1055–62.

2. Heemskerk-Gerritsen BAM, Jager A, Koppert LB, Obdeijn AIM, Collée M, Meijers-Heijboer HEJ, et al. Survival after bilateral risk-reducing mastectomy in healthy BRCA1 and BRCA2 mutation carriers. Breast Cancer Res Treat [Internet]. 2019 Oct 1 [cited 2024 Dec 17];177(3):723–33. Available from: https://pubmed.ncbi.nlm.nih.gov/31302855/

3. Bernstein-Molho R, Kaufman B, Ben David MA, Sklair-Levy M, Feldman DM, Zippel D, et al. Breast cancer surveillance for BRCA1/2 mutation carriers - is “early detection” early enough? Breast [Internet]. 2020 Feb 1 [cited 2024 Dec 17];49:81–6. Available from: https://pubmed.ncbi.nlm.nih.gov/31760168/

4. Frey JD, Salibian AA, Karp NS, Choi M. Comparing Therapeutic versus Prophylactic Nipple-Sparing Mastectomy: Does Indication Inform Oncologic and Reconstructive Outcomes? Plast Reconstr Surg. 2018 Aug;142(2):306–15.

5. Spear SL, Schwarz KA, Venturi ML, Barbosa T, Al-Attar A. Prophylactic mastectomy and reconstruction: clinical outcomes and patient satisfaction. Plast Reconstr Surg. 2008 Jul;122(1):1–9.

6. Isik A, Soran A, Grasi A, Barry N, Sezgin E. Lymphedema After Sentinel Lymph Node Biopsy: Who Is at Risk? Lymphat Res Biol. 2022 Apr;20(2):160–3.

7. Wrone DA, Tanabe KK, Cosimi AB, Gadd MA, Souba WW, Sober AJ. Lymphedema after sentinel lymph node biopsy for cutaneous melanoma: a report of 5 cases. Arch Dermatol. 2000 Apr;136(4):511–4.

8. Gabai-Kapara E, Lahad A, Kaufman B, Friedman E, Segev S, Renbaum P, et al. Population-based screening for breast and ovarian cancer risk due to BRCA1 and BRCA2. Proc Natl Acad Sci U S A. 2014 Sep 30;111(39):14205–10.

9. Franceschini G, Di Leone A, Terribile D, Sanchez MA, Masetti R. Bilateral prophylactic mastectomy in BRCA mutation carriers: what surgeons need to know. Ann Ital Chir. 2019;90:1–2.

10. King MC, Wieand S, Hale K, Lee M, Walsh T, Owens K, et al. Tamoxifen and breast cancer incidence among women with inherited mutations in BRCA1 and BRCA2: National Surgical Adjuvant Breast and Bowel Project (NSABP-P1) Breast Cancer Prevention Trial. JAMA. 2001 Nov 14;286(18):2251–6.

11. Marcinkute R, Woodward ER, Gandhi A, Howell S, Crosbie EJ, Wissely J, et al. Uptake and efficacy of bilateral risk reducing surgery in unaffected female BRCA1 and BRCA2 carriers. J Med Genet. 2022 Feb;59(2):133–40.

12. Heemskerk-Gerritsen BAM, Rookus MA, Aalfs CM, Ausems MGEM, Collée JM, Jansen L, et al. Improved overall survival after contralateral risk-reducing mastectomy in BRCA1/2 mutation carriers with a history of unilateral breast cancer: a prospective analysis. Int J Cancer. 2015 Feb 1;136(3):668–77.

13. Evans DGR, Ingham SL, Baildam A, Ross GL, Lalloo F, Buchan I, et al. Contralateral mastectomy improves survival in women with BRCA1/2-associated breast cancer. Breast Cancer Res Treat. 2013 Jul;140(1):135–42.

14. Pierce LJ, Phillips KA, Griffith KA, Buys S, Gaffney DK, Moran MS, et al. Local therapy in BRCA1 and BRCA2 mutation carriers with operable breast cancer: comparison of breast conservation and mastectomy. Breast Cancer Res Treat. 2010 Jun;121(2):389–98.

15. Yadav S, Boddicker NJ, Na J, Polley EC, Hu C, Hart SN, et al. Contralateral Breast Cancer Risk Among Carriers of Germline Pathogenic Variants in ATM, BRCA1, BRCA2, CHEK2, and PALB2. J Clin Oncol [Internet]. 2023 Mar 20 [cited 2024 Dec 17];41(9):1703–13. Available from: https://pubmed.ncbi.nlm.nih.gov/36623243/

16. DiSilvestro P, Banerjee S, Colombo N, Scambia G, Kim BG, Oaknin A, et al. Overall Survival With Maintenance Olaparib at a 7-Year Follow-Up in Patients With Newly Diagnosed Advanced Ovarian Cancer and a BRCA Mutation: The SOLO1/GOG 3004 Trial. J Clin Oncol. 2023 Jan 20;41(3):609–17.

17. Ray-Coquard I, Pautier P, Pignata S, Pérol D, González-Martín A, Berger R, et al. Olaparib plus Bevacizumab as First-Line Maintenance in Ovarian Cancer. N Engl J Med [Internet]. 2019 Dec 19 [cited 2024 Dec 17];381(25):2416–28. Available from: https://pubmed.ncbi.nlm.nih.gov/31851799/

18. John CS, Fong A, Alban R, Gillen J, Moore KM, Walsh CS, et al. Breast cancer surveillance following ovarian cancer in BRCA mutation carriers. Gynecol Oncol. 2022 Jan;164(1):202–7.

19. Nañez A, Stram DA, Bethan Powell C, Garcia C. Breast cancer risk in BRCA mutation carriers after diagnosis of epithelial ovarian cancer is lower than in carriers without ovarian cancer. Gynecol Oncol Rep. 2022 Feb;39:100899.

20. Safra T, Waissengrin B, Gerber D, Bernstein-Molho R, Klorin G, Salman L, et al. Breast cancer incidence in BRCA mutation carriers with ovarian cancer: A longitudal observational study. Gynecol Oncol. 2021 Sep;162(3):715–9.

21. Rebbeck TR, Lynch HT, Neuhausen SL, Narod SA, van’t Veer L, Garber JE, et al. Prophylactic Oophorectomy in Carriers of BRCA1 or BRCA2 Mutations . New England Journal of Medicine [Internet]. 2002 May 23 [cited 2024 Oct 6];346(21):1616–22. Available from: https://www.nejm.org/doi/full/10.1056/NEJMoa012158

22. Kotsopoulos J, Gronwald J, Huzarski T, Møller P, Pal T, McCuaig JM, et al. Bilateral Oophorectomy and All-Cause Mortality in Women With BRCA1 and BRCA2 Sequence Variations. JAMA Oncol [Internet]. 2024 Apr 18 [cited 2024 Jun 21];10(4):484–92. Available from: https://pubmed.ncbi.nlm.nih.gov/38421677/

23. Kuchenbaecker KB, Hopper JL, Barnes DR, Phillips KA, Mooij TM, Roos-Blom MJ, et al. Risks of breast, ovarian, and contralateral breast cancer for BRCA1 and BRCA2 mutation carriers. JAMA - Journal of the American Medical Association [Internet]. 2017 Jun 20 [cited 2021 Feb 18];317(23):2402–16. Available from: https://jamanetwork.com/

24. Menon U, Gentry-Maharaj A, Burnell M, Singh N, Ryan A, Karpinskyj C, et al. Ovarian cancer population screening and mortality after long-term follow-up in the UK Collaborative Trial of Ovarian Cancer Screening (UKCTOCS): a randomised controlled trial. Lancet [Internet]. 2021 Jun 5 [cited 2024 Dec 17];397(10290):2182–93. Available from: https://pubmed.ncbi.nlm.nih.gov/33991479/

25. Matan LS, Perri T, Kogan L, Brandt B, Meyer R, Levin G. Ovarian cancer risk management in BRCA-mutation carriers: A comparison of six international and national guidelines. Eur J Obstet Gynecol Reprod Biol [Internet]. 2022 Nov 1 [cited 2024 Dec 17];278:166–71. Available from: https://pubmed.ncbi.nlm.nih.gov/36208522/

26. Iavazzo C, Gkegkes ID, Vrachnis N. Primary peritoneal cancer in BRCA carriers after prophylactic bilateral salpingo-oophorectomy. J Turk Ger Gynecol Assoc [Internet]. 2016 Jun 1 [cited 2024 Dec 17];17(2):73–6. Available from: https://pubmed.ncbi.nlm.nih.gov/27403072/

27. Gasparri ML, Di Micco R, Zuber V, Taghavi K, Bianchini G, Bellaminutti S, et al. Ovarian reserve of women with and without BRCA pathogenic variants: A systematic review and meta-analysis. Breast. 2021 Dec;60:155–62.

28. Shapira M, Raanani H, Feldman B, Srebnik N, Dereck-Haim S, Manela D, et al. BRCA mutation carriers show normal ovarian response in in vitro fertilization cycles. Fertil Steril [Internet]. 2015 Nov 1 [cited 2024 Dec 17];104(5):1162–7. Available from: https://pubmed.ncbi.nlm.nih.gov/26335130/

29. Gunnala V, Fields J, Irani M, D’Angelo D, Xu K, Schattman G, et al. BRCA carriers have similar reproductive potential at baseline to noncarriers: comparisons in cancer and cancer-free cohorts undergoing fertility preservation. Fertil Steril [Internet]. 2019 Feb 1 [cited 2024 Dec 17];111(2):363–71. Available from: https://pubmed.ncbi.nlm.nih.gov/30527950/

30. Perri T, Naor-Revel S, Eliassi-Revivo P, Lifshitz D, Friedman E, Korach J. Fertility treatments and breast cancer risk in Jewish Israeli BRCA mutation carriers. Fertil Steril [Internet]. 2021 Aug 1 [cited 2024 Dec 17];116(2):538–45. Available from: https://pubmed.ncbi.nlm.nih.gov/33823990/

31. Liu X, Yue J, Pervaiz R, Zhang H, Wang L. Association between fertility treatments and breast cancer risk in women with a family history or BRCA mutations: a systematic review and meta-analysis. Front Endocrinol (Lausanne) [Internet]. 2022 Sep 13 [cited 2024 Dec 17];13. Available from: https://pubmed.ncbi.nlm.nih.gov/36176466/

32. Cullinane C, Gillan H, Geraghty J, Evoy D, Rothwell J, Mccartan D, et al. Fertility treatment and breast-cancer incidence: meta-analysis. BJS Open [Internet]. 2022 Feb 1 [cited 2024 Dec 17];6(1). Available from: https://pubmed.ncbi.nlm.nih.gov/35143625/

33. Van Bommel MHD, Inthout J, Veldmate G, Kets CM, De Hullu JA, Van Altena AM, et al. Contraceptives and cancer risks in BRCA1/2 pathogenic variant carriers: a systematic review and meta-analysis. Hum Reprod Update [Internet]. 2023 Mar 1 [cited 2024 Dec 17];29(2):197–217. Available from: https://pubmed.ncbi.nlm.nih.gov/36383189/

34. Moorman PG, Havrilesky LJ, Gierisch JM, Coeytaux RR, Lowery WJ, Urrutia RP, et al. Oral contraceptives and risk of ovarian cancer and breast cancer among high-risk women: a systematic review and meta-analysis. J Clin Oncol [Internet]. 2013 Nov 20 [cited 2024 Dec 17];31(33):4188–98. Available from: https://pubmed.ncbi.nlm.nih.gov/24145348/

35. Fitzpatrick D, Pirie K, Reeves G, Green J, Beral V. Combined and progestagen-only hormonal contraceptives and breast cancer risk: A UK nested case–control study and meta-analysis. PLoS Med [Internet]. 2023 Mar 1 [cited 2024 Dec 17];20(3):e1004188. Available from: https://journals.plos.org/plosmedicine/article?id=10.1371/journal.pmed.1004188

36. Gierach GL, Pfeiffer RM, Patel DA, Black A, Schairer C, Gill A, et al. Long-term overall and disease-specific mortality associated with benign gynecologic surgery performed at different ages. Menopause. 2014 Jun;21(6):592–601.

37. Li S, Silvestri V, Leslie G, Rebbeck TR, Neuhausen SL, Hopper JL, et al. Cancer Risks Associated With BRCA1 and BRCA2 Pathogenic Variants. J Clin Oncol [Internet]. 2022 May 10 [cited 2024 Dec 17];40(14):1529–41. Available from: https://pubmed.ncbi.nlm.nih.gov/35077220/

38. Oh M, McBride A, Yun S, Bhattacharjee S, Slack M, Martin JR, et al. BRCA1 and BRCA2 Gene Mutations and Colorectal Cancer Risk: Systematic Review and Meta-analysis. J Natl Cancer Inst. 2018 Nov 1;110(11):1178–89.

39. Breast Cancer Linkage Consortium. Cancer risks in BRCA2 mutation carriers. J Natl Cancer Inst. 1999 Aug 4;91(15):1310–6.

40. Moran A, O’Hara C, Khan S, Shack L, Woodward E, Maher ER, et al. Risk of cancer other than breast or ovarian in individuals with BRCA1 and BRCA2 mutations. Fam Cancer. 2012 Jun;11(2):235–42.

41. Lammert J, Grill S, Kiechle M. Modifiable Lifestyle Factors: Opportunities for (Hereditary) Breast Cancer Prevention - a Narrative Review. Breast Care (Basel). 2018 Apr;13(2):109–14.
