# Supplementary material for: Managing healthcare for female BRCA carriers in the population screening era: developing a harmonized national policy for surveillance and risk-reduction
Source: Isr J Health Policy Res. 2026 Jan 30;15:4. doi: 10.1186/s13584-026-00746-3 (PMC12857140; doi:10.1186/s13584-026-00746-3)
Supplement: Supplementary file 2 — Supplementary Material 2 [file 13584_2026_746_MOESM2_ESM.docx]

**Supplement 2- ESMO clinical practical guidelines grades of recommendation***


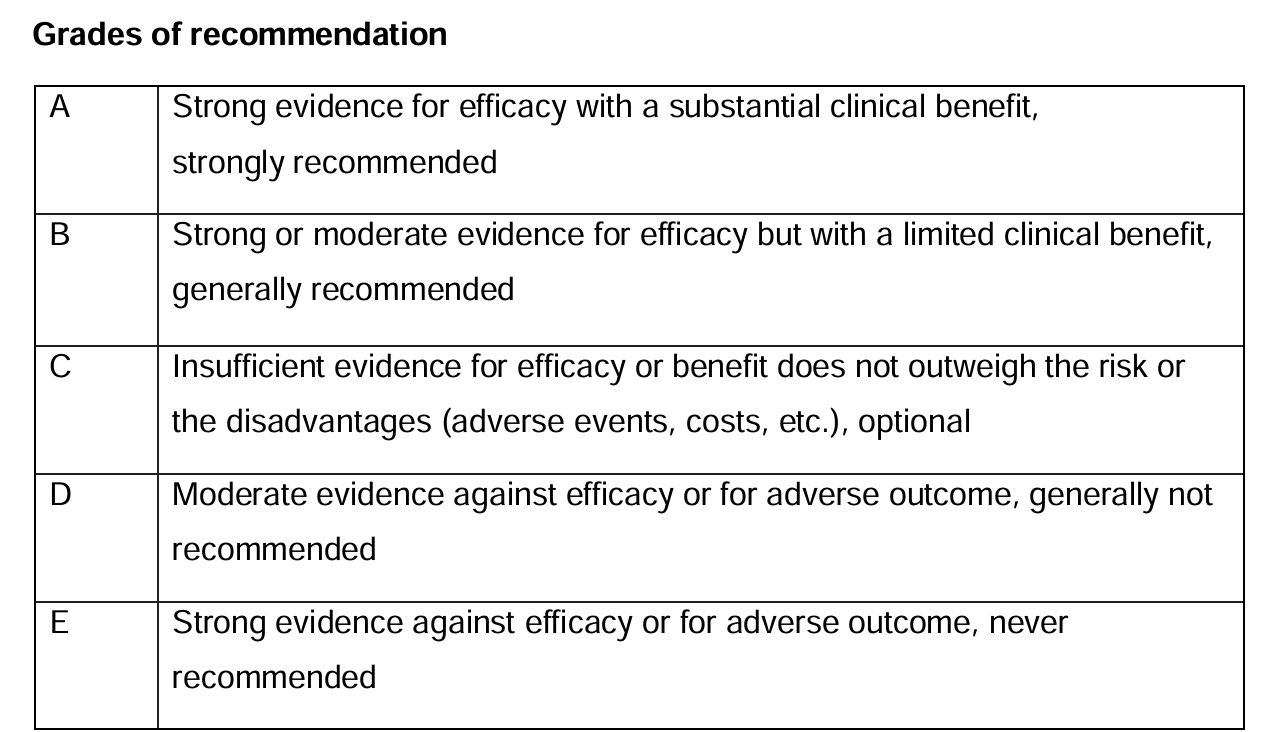


*Reprinted by permission of Oxford University Press on behalf of the Infectious Diseases Society of America^1,2^

**References:**

Dykewicz CA. Summary of the guidelines for preventing opportunistic infections among hematopoietic stem cell transplant recipients. Clin Infect Dis. 2001;33(2):139-144.

Gross PA, Barrett TL, Dellinger EP, et al. Purpose of quality standards for infectious diseases. Infectious Diseases Society of America. Clin Infect Dis. 1994;18(3):421.
